# Feasibility and Acceptability of Telephone-Administered Traumatic Brain Injury Common Data Elements and the Rehabilitation Needs Survey in Community-Dwelling Adults Exposed to the United States Criminal Legal System

**DOI:** 10.1089/neur.2024.0158

**Published:** 2025-03-26

**Authors:** Casey LaDuke, Kabrianna Tamura, Micaela Linder, Ronald Day, Kristen Dams-O’Connor

**Affiliations:** ^1^ Department of Psychology, John Jay College of Criminal Justice and The Graduate Center, City University of New York, New York, New York, USA.; ^2^ Brain Injury Research Center, Department of Rehabilitation and Human Performance, Icahn School of Medicine at Mount Sinai, New York, New York, USA.; ^3^ The Fortune Society, New York, New York, USA.; ^4^ Department of Neurology, Icahn School of Medicine at Mount Sinai, New York, New York, USA.

**Keywords:** brain injury, cognitive functioning, criminal justice, needs assessment, screening

## Abstract

Traumatic brain injury (TBI) is common among legally-impacted adults and has also been linked to negative outcomes throughout the criminal legal system. Despite this, relatively limited TBI research has focused on or even included legally-impacted adults. Existing literature in this population has used heterogeneous TBI definitions, populations, and measures when studying outcomes. This study therefore investigates the feasibility and acceptability of telephone-administered TBI common data elements (CDEs) and the Rehabilitation Needs Survey (RNS) in 85 legally-impacted community-dwelling adults. Regarding feasibility, completion rates across measures were high (88–100%), and noncompletion was most commonly due to participants declining to continue. Regarding acceptability, data collectors were able to administer and code most measures with relative ease. Reported difficulties related to measures requiring detailed data collector training to administer and code, such as the Brain Injury Screening Questionnaire (BISQ) and Brief Test of Adult Cognition by Telephone, or challenges inherent to self-report tests in general. In addition, data collectors recommended adding specific questions to the BISQ to query head injuries experienced during or as a result of their exposure to the criminal legal system. Overall, results support the use of telephone-administered TBI CDEs and the RNS in legally-impacted adults, and underscore the need for culturally-responsive training and technical assistance for TBI researchers engaging with this population.

## Introduction

Adults living with traumatic brain injury (TBI) and who have been exposed to the criminal legal system (hereinafter “legally-impacted adults”) represent a large and underserved group. The estimated lifetime prevalence of TBI in legally-impacted adults is 45.8% (95% confidence interval 37.8 − 54.1%),^1^ compared to 12.1% in the general population.^2^ With over 5 million people incarcerated in prisons and jails or under supervision in parole or probation in the United States,^3^ these estimates suggest there are several million people living with TBI and related needs in the United States criminal legal system alone.

Unfortunately, relatively limited systematic TBI research has centered on legally-impacted adults. This is likely due in part to the various ethical, clinical, and practical challenges to conducting clinical research with legally-impacted adults,^4,5^ the limited evidence base directly supporting the use of common TBI research methods in this population,^6,7^ and unfamiliarity among TBI researchers with population-relevant research considerations. This is an important gap, particularly given the high rates of TBI in legally-impacted adults,^1^ and preliminary evidence that TBI is a risk factor for poorer outcomes throughout the criminal legal system, including arrest,^8,9^ conviction,^10^ and incarceration;^11^ behavioral health difficulties and higher service utilization in correctional institutions;^12–17^ and lower treatment seeking/completion,^16^ and higher recidivism,^13,18,19^ following release into the community. Furthermore, this preliminary research has been heterogeneous in the methods used to measure and define TBI itself, relevant covariates, and related outcomes. As a result, fundamental questions about the characteristics and needs of legally-impacted adults with TBI remain unanswered.

This study takes significant steps toward addressing these concerns by investigating the feasibility (completion rates) and acceptability (data collector perceptions)^20^ of several telephone-administered screening tools selected from the TBI common data elements (CDEs)^21^ and the Rehabilitation Needs Survey (RNS)^22^ with legally-impacted adults living in the community. The TBI CDEs were developed to standardize the definition and collection of TBI data and support scientific rigor, data sharing, and collaboration in TBI research.^21^ The recently developed RNS is a validated TBI needs assessment tool^22^ that addresses an important gap in the current TBI CDEs of direct relevance to historically underserved populations.

To the best of our knowledge, this study is the first systematic investigation of the selected TBI CDEs and RNS in legally-impacted adults. Establishing their feasibility and acceptability will advance TBI research with this population by promoting the use of these rigorous methods in future studies centering on legally-impacted adults, and by supporting the inclusion of legally-impacted adults in ongoing systematic research already using these methods. This, in turn, will enable meaningful comparisons of findings across studies. This study also appears to be the first to leverage the benefits of telephone administration of rigorous TBI screening tools (especially TBI history/severity^23^ and cognition^24,25^) in a sample of community-dwelling adults with various exposures to the United States criminal legal system. These benefits include accessibility, scalability, and efficient use of resources, which may be particularly useful in research with legally-impacted adults given the large size and geographic diversity of this group, and the restricted access and resource limitations inherent to those incarcerated in prisons or jails. If successful, this study will support the implementation of these rigorous TBI screening methods to better understand and address the needs of this large, diverse, and underserved TBI group.

## Materials and Methods

### Participants

Participants were adults served by The Fortune Society (Fortune) in New York City, NY, USA. Founded in 1967, Fortune (fortunesociety.org) serves thousands of individuals each year using a holistic one-stop model to provide a broad range of diversion programs (i.e., diverting people from the criminal legal system and into appropriate services), re-entry programs (i.e., providing services to people re-entering society from the criminal legal system), medical and behavioral health care, education and employment training, and other services. Exclusion criteria were being under age 18, self-reported physical or sensory difficulties that interfered with using a telephone, self-reported or observed difficulties with English that precluded them from engaging with study procedures, or a self-reported or observed medical status (e.g., psychiatric crisis, intoxication, and acute withdrawal) that precluded them from safely engaging in research.

### Procedures

This study was reviewed and approved by the City University of New York Human Research Protection Program (#2021-0337). Data for this study were collected between November 2021 and May 2022. Participants were recruited in person during their regular engagement in services at Fortune, during which they were introduced to the study and completed a screening of exclusion criteria. Eligible participants then completed scheduling, informed consent, data collection, debriefing, and compensation by telephone. All procedures were completed in English. Data collection was estimated to take 90 min and participants were compensated $75 for their participation.

Measures were generally administered and scored according to their standardized instructions (see *Measures* section for relevant modifications). Participants were asked to sit in a quiet environment and not use writing or other recording materials. Data collectors were instructed to provide passive encouragement and discontinue any study procedure as they deemed necessary. Potentially invalid results were flagged by data collectors for further review by the first author (C.L.). Measure completion (i.e., administered in full), validity (i.e., results valid or invalid), and reasons for noncompletion (i.e., attempted but not completed in full) were recorded (see Table 1 for a summary of codes and relevant definitions). These data will be used to assess *feasibility*, defined here as the extent to which the materials and methods can be successfully used or carried out with consumers (i.e., participants) in this setting.^20^

**Table 1.** Test Completion Codes

Code	Description
Test administered in full:	
01	Results valid
02	Results pending^a^
03	Results invalid
Test attempted but not completed, due to:	
11	Cognitive/neurological reasons^b^
12	Other medical reasons^b^
13	Interactive complexity^c^
14	Participant declined to continue
15	Interrupted administration^d^
16	Other logistical reasons
17	Test not attempted
20	Other

^a^ Used for internal purposes only; reviewed and updated to relevant code in the final dataset.

^b^ Symptom severity that precluded measurement completion.

^c^ Data collection being discontinued due to complications with participants’ communication or behavior.

^d^ Technological interruptions, scheduling conflicts, or similar logistical reasons without re-engaging in data collection.

One data collector (C.L.) earned a doctoral degree in clinical psychology and had extensive training and experience in clinical interviewing and cognitive testing, including with legally-impacted adults. Two data collectors (including K.T.) were recruited from doctoral programs in clinical psychology, had previously earned master’s degrees in forensic psychology or criminal justice (respectively), and had prior training and experience with clinical interviewing and cognitive testing, including legally-impacted adults. The remaining four data collectors were recruited from master’s programs in forensic psychology or forensic mental health counseling and had training in clinical interviewing and cognitive testing (including with legally-impacted adults) but little to no experience.

The first author (C.L.) oversaw the initial training of all other data collectors, including asynchronous review of study materials and supporting literature followed by live or recorded trainings addressing study procedures and measures. The first and second authors (C.L. and K.T.) oversaw further practical training of all other data collectors, including the review, coding, and entry of three recorded data collection sessions (one mock and two actual) followed by individual feedback and retrainings as needed.

Several quality assurance methods were instituted to ensure methodological fidelity. These included de-identified recording of data collection sessions (with consent) to allow for independent reviews as needed and double-coding whenever possible, 100% double entry, and periodic retrainings as needed. In addition, data dictionaries and standard operating procedures were developed to guide data collection, entry, and cleaning, and were further supported by regular teleconferences, an email listserv, and external technical support as needed.

At the end of their involvement, all data collectors (*N* = 7) were invited to complete an online survey developed for this study to investigate their experiences. These data will be used to assess *acceptability*, defined here as the perceptions among implementation stakeholders (i.e., data collectors) that the materials and methods were agreeable, palatable, or satisfactory.^20^

### Measures

All standardized measures and related subtests are summarized and described in Table 2. Several standardized and other measures requiring further description are presented below.

**Table 2.** Summary and Description of Study Measures/Subtests

Measure/subtest	Description
FITBIR Demographics Form^*^	Standardized screening of demographic information developed for use across TBI research projects.^a^
Brief Test of Adult Cognition by Telephone (BTACT)^*^	Telephone-administered cognitive test battery measuring the following constructs (and domains):
Word List Recall (RAVLT)	Episodic verbal memory (memory)
Short-Delay Recall (RAVLT)	Episodic verbal memory (memory)
Digits Backward (WAIS-III)	Working memory (executive functioning)
Category Fluency	Semantic verbal fluency (executive functioning)
Number Series (Reasoning Test)	Abstract reasoning (executive functioning)
Backward Counting	Processing speed (executive functioning)
Brain Injury Screening Questionnaire (BISQ)^*^	Structured screening tool of the lifetime history and severity of head trauma and TBI.
Post Discharge/Outpatient Treatment^*^	Screening measure of lifetime history of therapy and rehabilitation services following TBI.^b^
TBI Symptoms and Signs^*^	Screening measure of the presence of 20 specific symptoms and signs following TBI.^b^
Sensory screening^*^	Five single-item screening tools for the presence of specific symptoms related to balance, hearing, taste/smell, tinnitus, and speech following TBI.^b^
PTSD Checklist—Civilian Version (PCL-C)^*^	Standardized self-report rating scale for PTSD.
Behavioral History^*^	Screening measure of past and current use of alcohol, tobacco, and illicit substances.^b^
Satisfaction with Life Scale (SWLS)^*^	Standardized self-report rating scale for overall satisfaction with life.
Rehabilitation Needs Survey (RNS)	Standardized self-report measure to identify, quantify, and characterize rehabilitation service needs following TBI.

^*^ TBI Common Data Element.

^a^ Included Core and Supplemental items.

^b^ Developed specifically for the TBI Common Data Elements.

EF, executive functioning; FITBIR, Federal Interagency Traumatic Brain Injury Research; PTSD, Post-Traumatic Stress Disorder; RAVLT, Rey Auditory Verbal Learning Test; TBI, traumatic brain injury; WAIS-III, Weschler Adult Intelligence Scale–Third Edition.

#### Brief Test of Adult Cognition by Telephone

The Brief Test of Adult Cognition by Telephone (BTACT) is a telephone-administered cognitive test battery developed from several common neuropsychological tests encompassing a wide range of cognitive domains and ability levels.^24^ The subtests included in this study are summarized in Table 2. The Stop and Go Switch Task was not included as it is not required for composite scores similar to those used in prior BTACT validation research in TBI populations,^25^ and due to potential confounds when measuring reaction time by telephone.^26^

#### Brain Injury Screening Questionnaire

The Brain Injury Screening Questionnaire (BISQ) is a structured screening tool for the lifetime history and severity of head trauma exposures and TBI.^23^ Participants are asked whether they have ever experienced a blow to the head in 19 specific situations, and an open-ended item to report head trauma in situations not queried. Supplemental items query whether they have ever been hospitalized or treated in an emergency department for eight specific medical events (plus any other self-reported event) to document alternative contributors to clinical symptoms. Each endorsed item is followed by additional questioning to determine the number of blows to the head sustained, the presence and length of any periods of loss of consciousness and alterations of consciousness, and the date of each occurrence. The version used in this study also included three modules to assess head injury exposure due to sports, military activity, and intimate partner violence.

#### Post-Traumatic Stress Disorder Checklist—Civilian Version

The Post-Traumatic Stress Disorder (PTSD) Checklist—Civilian Version (PCL-C) is a standardized self-report rating scale for PTSD).^27^ Participants were presented with 17 items corresponding to key PTSD symptoms and asked how much they have been bothered by each item in the last month on a scale of 1 (not at all) to 5 (extremely).

#### Satisfaction with Life Scale

The Satisfaction with Life Scale (SWLS) is a standardized self-report rating scale for overall satisfaction with life.^28^ Participants are presented with five statements and asked to indicate their agreement on a scale of 1 (strongly disagree) to 7 (strongly agree).

#### Rehabilitation Needs Survey

The Rehabilitation Needs Survey (RNS) is a standardized self-report measure to identify, quantify, and characterize rehabilitation service needs following TBI.^22^ Participants are presented with 21 rehabilitation needs common following TBI, and asked (a) whether they have ever received help in that area and (b) whether they need or want (more) help. Each item is then categorized as a met need, partially met need, unmet need, or not a need. RNS administration was modified by adapting Item 21 to “Adjusting to life in the community” (vs. “Transitioning from military to civilian life”) to approximate the underlying need in this sample.

#### Data Collectors Survey

A survey was developed to investigate data collectors’ experiences with this study (available in Supplementary Data S1). Core items surveyed perceptions about the administration and coding of 44 aspects of the standard assessment protocol, on a scale of 1 (extremely difficult) to 5 (extremely easy). Supplemental items surveyed perceptions about each measure/subtest and other study procedures.

### Statistical analysis

Descriptive analyses were performed for sample demographics and completion rates. Responses to the data collector survey were tabulated and grouped by study measure/subtest. Quantitative analyses were performed using IBM SPSS 29. Qualitative responses to the data collector survey were reviewed by the first author (C.L.) using an open coding framework, which is an exploratory and inductive method designed to find meaningful results close to the data itself (i.e., versus existing theories).

## Results

Eighty-five individuals participated in this study. Sample demographics and criminal legal exposures are summarized in Table 3.

**Table 3.** Summary of Sample Demographics and Criminal Legal Exposures

Demographics (*N* = 85)	M	SD	*n*	%
Age, year	43.8	12.0		
Gender				
Male			68	80
Female			17	20
Racial background				
American Indian or Alaska Native			10	12
Asian			3	4
Black or African-American			60	71
Native Hawaiian or other Pacific Islander			0	0
White			12	14
Unknown			9	11
Not reported			0	0
Ethnic background				
Hispanic or Latino			22	26
Not Hispanic or Latino			48	57
Other^a^			20	24
Unknown			0	0
Not reported			1	1

Criminal legal exposures (*N* = 83)	*n*	%	Mdn (IQR)	Range
Current (# yes)				
Parole or other supervised release	25	30		
Probation	5	6		
Alternative-to-incarceration program	21	25		
Prior (# yes)				
Prison	55	66		
Jail	61	74		
Parole	35	42		
Probation	28	34		
Alternative-to-incarceration program	27	33		
Total lifetime exposures (total #)				
Prison			1 (0–3)	0–70
Jail			2 (0–5.25)	0–118
Parole			1 (0–2)	0–6
Probation			0 (0–1)	0–3
Alternative-to-incarceration program			0 (0–1)	0–5

Demographic variable labels and response options defined by Federal Interagency Traumatic Brain Injury Research (FITBIR) Demographics Form. Percentages are rounded to the nearest integer.

^a^ Included responses commonly defined as racial backgrounds (e.g., Black, Native American, White; *n* = 9), specific countries (*n* = 7), and other self-defined ethnic backgrounds (*n* = 1).

IQR, interquartile range.

### Feasibility

Rates of completion and reasons for noncompletion are shown in Tables 4 and 5. Overall, a large majority of participants completed and provided valid results for all TBI CDEs (88–100%) and the RNS (92%). Invalid results were not observed for any measure (0%). Non-completion was rare, and mostly due to participants declining to continue following an individual measure or item (1–5% on 6/10 TBI CDEs; 0% on RNS) followed by interactive complexity, interrupted administration, or other logistical reasons (all 1% on 2/10 TBI CDEs; 0% on RNS). Non-completion due to cognitive/neurological or other medical reasons was not observed in this sample (0%). Non-attempt was also rare (5–9% on 7/10 TBI CDEs; 8% on RNS) and due exclusively to data collection having been discontinued prior to the attempt rather than anything related to these measures themselves. Other codes were also rare (1–2% on 5/10 TBI CDEs). Similar results were found for the BTACT subtests in terms of completion (95–100% complete and valid; 1% complete but invalid on 4/6 subtests), non-completion (1% of the participants declined to continue on 3/6 subtests), and non-attempt (2% limited only to the last subtest administered).

**Table 4.** Sample Completion Codes

		Test attempted but not completed, due to:					
	Test administered in full—results valid	Interactive complexity	Participant declined to continue	Interrupted administration	Other logistical reasons	Test not attempted	Other
FITBIR Demographics^a^	100%						
BTACT	95%		1%		1%		2%
BISQ	95%	1%	1%				2%
Post Discharge/Outpatient Treatment	93%					5%	2%
TBI Symptoms and Signs	95%					5%	
Sensory screening	93%					5%	2%
PCL-C	93%		1%			5%	1%
Behavioral History	91%		4%	1%		5%	
RNS	92%					8%	
SWLS	88%		1%		1%	9%	
FITBIR Demographics^b^	89%	1%	5%	1%		4%	

*N* = 85. Measures organized by standardized order of administration. Completion codes without responses were excluded, specifically: test administered in full—results invalid; test attempted but not completed, due to cognitive/neurological reasons; and test attempted but not completed, due to other medical reasons (all *n* = 0, 0%). Cells without responses (0%) were left blank for clarity. Percentages are rounded to the nearest integer.

^a^ Core items.

^b^ Supplemental items.

BTACT, Brief Test of Adult Cognition by Telephone; BISQ, Brain Injury Screening Questionnaire; FITBIR, Federal Interagency Traumatic Brain Injury Research; PCL-C, Post-Traumatic Stress Disorder (PTSD) Checklist—Civilian Version; RNS, Rehabilitation Needs Survey; SWLS, Satisfaction with Life Scale; TBI, traumatic brain injury.

**Table 5.** Brief Test of Adult Cognition by Telephone Subtest Completion Codes

	Test administered in full		Test attempted but not completed, due to participant declining to continue	Test not attempted
	Results valid	Results invalid		
Word List Recall	99%	1%		
Digits Backward	98%	1%	1%	
Category Fluency	100%			
Number Series	99%		1%	
Backward Counting	99%	1%		
Short-Delay Word Recall	95%	1%	1%	2%

*N* = 85. Subtests are organized by standardized order of administration. Completion codes without responses were excluded, specifically: Test attempted but not completed, due to cognitive/neurological reasons, other medical reasons, interactive complexity, interrupted administration, or other logistical reasons; and other (all *n* = 0, 0%). Cells without responses (0%) were left blank for clarity. Percentages are rounded to the nearest integer.

### Acceptability

Five of seven data collectors participated in the acceptability survey (71% response rate). This included data collectors who had experience with the study measures and procedures via direct administration and coding (*n* = 2), review and data cleaning (*n* = 1), or both (*n* = 2 including the first and second authors). A summary of data collectors’ perceptions regarding the acceptability of the measures and subtests is shown in Table 6. Overall, little to no difficulties were reported for nearly all of the TBI CDEs or the RNS. Areas of relative difficulty noted by data collectors are summarized below.

**Table 6.** Data Collectors’ Perceptions Regarding Acceptability of Measures and Subtests

	Administration			Coding		
	Mdn	Mode	Diff.*^a^*	Mdn	Mode	Diff.*^a^*
FITBIR Demographics						
Core	5	5	0%			
Supplemental	3.5	4	20%			
BTACT						
Word List Recall	5	5	0%	5	5	0%
Digits Backward	4	4	0%	5	5	0%
Category Fluency	4	5	20%	4	2	40%
Number Series	3	2	40%	5	5	0%
Backward Counting	3	5	40%	2	1	80%
Short-Delay Word Recall	5	5	0%	5	5	0%
BISQ						
HIE: Participation in organized sports	4	3	0%			
HIE: Blows to the head from sports	2	2	60%			
HIE: Military activity	5	5	0%			
HIE: Blows to the head from IPV	4	4	40%			
BISQ: Other Injuries to the head	3	3	20%			
Injury experience				3	3	20%
Injury count				2	2	60%
Hospitalization/ER experience				3	2	40%
Hospitalization/ER count				2	2	60%
Loss of consciousness				2	2	60%
Alteration of consciousness				2	2	60%
Date(s) of occurrence				1	1	100%
Post Discharge/Outpatient Treatment	4	4	0%			
TBI Symptoms and Signs	5	5	0%			
Sensory screening	5	5	0%			
PCL-C	4	4	20%	4	4	0%
Behavioral History	2	2	80%	3	3	20%
RNS	4	4	0%			
SWLS	5	5	0%	5	5	0%

*N* = 5. Measures/subtests are organized by standardized order of administration. Ratings are summarized by median (Med) and mode, based on a scale of 1 (extremely difficult) to 5 (extremely easy).

^a^ Percent of data collectors reporting difficulty, based on combined ratings of 1 (extremely difficult) or 2 (somewhat difficult).

BTACT, Brief Test of Adult Cognition by Telephone; BISQ, Brain Injury Screening Questionnaire; FITBIR, Federal Interagency Traumatic Brain Injury Research; HIE, head injury exposure module; IPV, intimate partner violence; PCL-C, Post-Traumatic Stress Disorder (PTSD) Checklist—Civilian Version; RNS, Rehabilitation Needs Survey; SWLS, Satisfaction with Life Scale; TBI, traumatic brain injury.

On the BISQ, data collectors reported that some participants struggled to recall dates of injury, number of blows to the head sustained, and details required to ascertain the duration of both loss and alteration of consciousness. Qualitatively, data collectors reported that some participants found the BISQ somewhat long and tedious, especially those with extensive TBI history. In addition, data collectors reported that some participants had particular difficulty reporting event-specific information for events that occurred in childhood. Last, all data collectors suggested that it would be useful to add item(s) specific to hits to the head directly related to their exposure to the criminal legal system when working with this population to better capture TBI history, severity, and related context.

Some administration challenges were reported for three BTACT subtests, especially Backward Counting but also Category Fluency and Number Series. In particular, data collectors reported difficulty balancing the dual demands of timing and recording responses and therefore found audio recording helpful to ensure the accuracy of scoring.

Last, some administration challenges were reported for the Behavioral History measure. Qualitatively, data collectors reported some participants had difficulty quantifying substance use (especially alcohol) on this measure, and reported that item wording was occasionally confusing to participants and may have led to inaccurate responses (e.g., licit versus illicit use of medications). Data collectors noted that the inclusion of marijuana in illicit drugs posed challenges in the context of jurisdictional trends toward decriminalization.

## Discussion

This article presented a protocol for implementing telephone-administered TBI CDEs and the RNS in 85 community-dwelling adults with prior and ongoing exposures to the United States criminal legal system, and provided evidence for their feasibility and acceptability in this important TBI group. Participants were generally able to complete and provide valid results for the selected measures. In addition, data collectors were generally able to administer and code the selected measures with relative ease, with difficulties mostly reported for measures requiring relatively higher levels of training to administer and code (e.g., BISQ and BTACT) or self-report of details people commonly find challenging (e.g., dates of head injuries sustained in groups with high levels of violence exposure, and quantitative amounts of alcohol consumption). Last, data collectors unanimously recommended that the BISQ (and similar TBI screening measures) include items specific to head injuries experienced during or as a result of their exposures to the criminal legal system to more fully capture TBI history in this population.

TBI history/severity and cognitive functioning are foundational to contemporary TBI research, and any heightened efforts related to training, technical assistance, and quality assurance related to such measures (e.g., the BISQ and BTACT) are clearly worthwhile. This appears particularly warranted with data collection conducted by groups less specialized in TBI research, such as the university-based data collectors involved in this study. Conversely, the high completion rates and general ease reported by data collectors in this study underscores the benefits of their training and experiences working with legally-impacted adults, including effective clinical interviewing in the context of the structure and practical realities of the criminal legal system, this population’s historical marginalization and oppression in and by clinical research, and related barriers in personal, social, and structural determinants of health. This finding highlights the need for culturally responsive training and technical assistance for TBI research groups engaging with legally-impacted adults.

There are several limitations that merit further discussion. The sample of legally-impacted adults was relatively small (*N* = 85), mostly male (80%), recruited from a single community site with a relatively high level of resources, and all resided in a large urban area in the northeastern United States, all of which may limit the generalizability of the presented feasibility evidence. Relatedly, all participants resided in the community and used their personal phones to complete the research protocol, limiting our ability to understand the feasibility and acceptability of these methods and measures with legally-impacted adults in correctional institutions. In addition, all data collectors were drawn from relevant graduate training programs, and none had prior research experience in TBI or with the selected measures. While this underscores the benefits and successful implementation of these standardized research methods in real-world settings, it may also limit the generalizability of the acceptability evidence (and possibly the feasibility evidence) to those better versed in TBI research but with less experience with this novel TBI population. Relatedly, several procedures were implemented to increase the quality of the data, though these may not be reasonably efficient (e.g., audio recording, double-coding, 100% double entry) or accessible in all settings (e.g., technical assistance from researchers specializing in people with TBI and legally-impacted adults, respectively). Last, the sample of data collectors providing their perceptions of the study was relatively small (*n* = 5), which may limit the interpretation of the related quantitative data and the generalizability of the acceptability evidence in general.

Feasibility and acceptability are foundational to the use of rigorous TBI research methods in the large and underserved population of legally-impacted adults. Further cross-validation of the telephone-administered TBI CDEs and recently developed standardized screen of related rehabilitation needs described here is still needed in this important TBI group. This is particularly true for the BTACT, as prior evidence suggests the cultural identities and clinical characteristics of legally-involved adults are poorly captured in the development and standardization of measures of cognition.^7,29^ Nonetheless, the selected TBI CDEs and RNS hold great promise to better understand the characteristics and needs of individuals with TBI in the United States criminal legal system, and the future development of related intervention strategies that are likely to have significant impacts on public health and safety.

## Abbreviations Used

CDEs: Common data elements
RNS: Rehabilitation Needs Survey
SWLS: Satisfaction with Life Scale
TBI: Traumatic brain injury
