# Supplementary material for: Feasibility and Acceptability of Telephone-Administered Traumatic Brain Injury Common Data Elements and the Rehabilitation Needs Survey in Community-Dwelling Adults Exposed to the United States Criminal Legal System
Source: Neurotrauma Rep. 2025 Mar 26;6(1):274–82. doi: 10.1089/neur.2024.0158 (PMC12040535; doi:10.1089/neur.2024.0158)
Supplement: Supplementary Data [file neur.2024.0158_supplementary_data.docx]

**Supplemental Data**

Data collectors survey

Thank you for taking the time to provide feedback for the Brain Health Project! The following pages contain several questions for each interview measure. If possible, please provide a response to each item based on your experience, in whatever format you prefer or level of detail you think is necessary. If you really cannot think of a response for any particular item, it is okay to leave that item blank.

Start of Block: Demographics

How would you rate the administration of the following Demographics measures?

|  | Extremely difficult (1) | Somewhat difficult (2) | Neither easy nor difficult (3) | Somewhat easy (4) | Extremely easy (5) |
| --- | --- | --- | --- | --- | --- |
| Core |  |  |  |  |  |
| Supplemental |  |  |  |  |  |

What do you like about the Demographics measure? [Open text response]

What do you dislike about the Demographics measure? [Open text response]

What changes (if any) do you think would improve the Demographics measure? [Open text response]

End of Block: Demographics

Start of Block: BTACT

When introducing the BTACT...

- Do participants have any specific difficulty with the general instructions? (1) __________________________________________________
- Are there certain parts of the instructions for any specific subtest that are commonly questioned, misunderstood, or unclear? (2) __________________________________________________
- Do you have strategies or keywords that you use to clear up these issues? (3) __________________________________________________

How do you distinguish between a language barrier, a situational barrier, and a cognitive barrier when administering and scoring the BTACT? [Open text response]

How do you distinguish between a cognitive barrier and a refusal? Can you provide specific anecdotes and/or describe how you determined a participant was unable/unwilling to proceed due to cognitive barrier or refused? [Open text response]

What do you do if a participant becomes distracted, or appears to be in a situation not fully suitable for cognitive testing (quiet, no distractions, good phone connection)? [Open text response]

How would you rate the administration of the BTACT subtests?

|  | Extremely difficult (1) | Somewhat difficult (2) | Neither easy nor difficult (3) | Somewhat easy (4) | Extremely easy (5) |
| --- | --- | --- | --- | --- | --- |
| Word List Recall (1) |  |  |  |  |  |
| Digits Backward (2) |  |  |  |  |  |
| Category Fluency (3) |  |  |  |  |  |
| Number Series (Reasoning Test) (4) |  |  |  |  |  |
| Backward Counting (5) |  |  |  |  |  |
| Short-Delay Word Recall (6) |  |  |  |  |  |

How often do participants appear to have problems with the following subtest instructions?

|  | Never (1) | Sometimes (2) | About half the time (3) | Most of the time (4) | Always (5) |
| --- | --- | --- | --- | --- | --- |
| Word List Recall (1) |  |  |  |  |  |
| Digits Backward (2) |  |  |  |  |  |
| Category Fluency (3) |  |  |  |  |  |
| Number Series (Reasoning Test) (4) |  |  |  |  |  |
| Backward Counting (5) |  |  |  |  |  |
| Short-Delay Word Recall (6) |  |  |  |  |  |

Is the subtest generally liked or disliked by participants?

|  | Disliked a great deal (1) | Disliked somewhat (2) | Neither liked nor disliked (3) | Liked somewhat (4) | Liked a great deal (5) |
| --- | --- | --- | --- | --- | --- |
| Word List Recall (1) |  |  |  |  |  |
| Digits Backward (2) |  |  |  |  |  |
| Category Fluency (3) |  |  |  |  |  |
| Number Series (Reasoning Test) (4) |  |  |  |  |  |
| Backward Counting (5) |  |  |  |  |  |
| Short-Delay Word Recall (6) |  |  |  |  |  |

How can you tell these subtests are liked/disliked?

- Word List Recall (1) __________________________________________________
- Digits Backward (2) __________________________________________________
- Category Fluency (3) __________________________________________________
- Number Series (Reasoning Test) (4) __________________________________________________
- Backward Counting (5) __________________________________________________
- Short-Delay Word Recall (6) __________________________________________________

Why do participants like or dislike this subtest?

- Word List Recall (1) __________________________________________________
- Digits Backward (2) __________________________________________________
- Category Fluency (3) __________________________________________________
- Number Series (Reasoning Test) (4) __________________________________________________
- Backward Counting (5) __________________________________________________
- Short-Delay Word Recall (6) __________________________________________________

For Word Recall (Recall and Short-Delay), some data collectors have indicated that if the participant says all of the words that they can recall within the first 10-15 seconds, and you cue them that they still “have time to try to recall more words,” that they get annoyed or anxious that they have to wait for the 90 seconds. How often did you experience this with participants?

- Never (1)
- Sometimes (2)
- About half the time (3)
- Most of the time (4)
- Always (5)

For Number Series (Reasoning Test), some data collectors have indicated that the concept of “okay” or providing some sort of affirmation before moving onto the next number is especially frustrating to participants. How often did you experience this with participants?

- Never (1)
- Sometimes (2)
- About half the time (3)
- Most of the time (4)
- Always (5)

How would you rate the scoring of the BTACT subtests?

|  | Extremely difficult (1) | Somewhat difficult (2) | Neither easy nor difficult (3) | Somewhat easy (4) | Extremely easy (5) |
| --- | --- | --- | --- | --- | --- |
| Word List Recall (1) |  |  |  |  |  |
| Digits Backward (2) |  |  |  |  |  |
| Category Fluency (3) |  |  |  |  |  |
| Number Series (Reasoning Test) (4) |  |  |  |  |  |
| Backward Counting (5) |  |  |  |  |  |
| Short-Delay Word Recall (6) |  |  |  |  |  |

What do you like about the BTACT? [Open text response]

What do you dislike about the BTACT? [Open text response]

What changes (if any) do you think would improve the BTACT for use with people involved in the criminal legal system? [Open text response]

End of Block: BTACT

Start of Block: BISQ

When introducing the BISQ...

- Do participants have any specific difficulty with the general instructions? (1) __________________________________________________
- Are there certain parts of the instructions for any specific section that are commonly questioned, misunderstood, or unclear? (2) __________________________________________________
- Do you have strategies or keywords that you use to clear up these issues? (3) __________________________________________________

How would you rate the administration of the BISQ sections?

|  | Extremely difficult (1) | Somewhat difficult (2) | Neither easy nor difficult (3) | Somewhat easy (4) | Extremely easy (5) |
| --- | --- | --- | --- | --- | --- |
| Head Injury Exposure: Participation in Organized Sports (1) |  |  |  |  |  |
| Head Injury Exposure: Blows to the Head from Sports (2) |  |  |  |  |  |
| Head Injury Exposure: Military Activity (3) |  |  |  |  |  |
| Head Injury Exposure: Blows to the Head from Intimate Partner Violence (4) |  |  |  |  |  |
| Other Injuries to the Head: Brain Injury Screening Questionnaire (5) |  |  |  |  |  |
| Hospitalizations: Brain Injury Screening Questionnaire (6) |  |  |  |  |  |
| Health Conditions (7) |  |  |  |  |  |
| Additional Questions (8) |  |  |  |  |  |

How often do participants appear to have problems completing the following sections?

|  | Never (1) | Sometimes (2) | About half the time (3) | Most of the time (4) | Always (5) |
| --- | --- | --- | --- | --- | --- |
| Head Injury Exposure: Participation in Organized Sports (1) |  |  |  |  |  |
| Head Injury Exposure: Blows to the Head from Sports (2) |  |  |  |  |  |
| Head Injury Exposure: Military Activity (3) |  |  |  |  |  |
| Head Injury Exposure: Blows to the Head from Intimate Partner Violence (4) |  |  |  |  |  |
| Other Injuries to the Head: Brain Injury Screening Questionnaire (5) |  |  |  |  |  |
| Hospitalizations: Brain Injury Screening Questionnaire (6) |  |  |  |  |  |
| Health Conditions (7) |  |  |  |  |  |
| Additional Questions (8) |  |  |  |  |  |

Are the BISQ sections generally liked or disliked by participants?

|  | Disliked a great deal (1) | Disliked somewhat (2) | Neither liked nor disliked (3) | Liked somewhat (4) | Liked a great deal (5) |
| --- | --- | --- | --- | --- | --- |
| Head Injury Exposure: Participation in Organized Sports (1) |  |  |  |  |  |
| Head Injury Exposure: Blows to the Head from Sports (2) |  |  |  |  |  |
| Head Injury Exposure: Military Activity (3) |  |  |  |  |  |
| Head Injury Exposure: Blows to the Head from Intimate Partner Violence (4) |  |  |  |  |  |
| Other Injuries to the Head: Brain Injury Screening Questionnaire (5) |  |  |  |  |  |
| Hospitalizations: Brain Injury Screening Questionnaire (6) |  |  |  |  |  |
| Health Conditions (7) |  |  |  |  |  |
| Additional Questions (8) |  |  |  |  |  |

How can you tell these BISQ sections are liked or disliked?

- Head Injury Exposure: Participation in Organized Sports (1) __________________________________________________
- Head Injury Exposure: Blows to the Head from Sports (2) __________________________________________________
- Head Injury Exposure: Military Activity (3) __________________________________________________
- Head Injury Exposure: Blows to the Head from Intimate Partner Violence (4) __________________________________________________
- Other Injuries to the Head: Brain Injury Screening Questionnaire (5) __________________________________________________
- Hospitalizations: Brain Injury Screening Questionnaire (6) __________________________________________________
- Health Conditions (7) __________________________________________________
- Additional Questions (8) __________________________________________________

Why do participants like or dislike these BISQ sections?

- Head Injury Exposure: Participation in Organized Sports (1) __________________________________________________
- Head Injury Exposure: Blows to the Head from Sports (2) __________________________________________________
- Head Injury Exposure: Military Activity (3) __________________________________________________
- Head Injury Exposure: Blows to the Head from Intimate Partner Violence (4) __________________________________________________
- Other Injuries to the Head: Brain Injury Screening Questionnaire (5) __________________________________________________
- Hospitalizations: Brain Injury Screening Questionnaire (6) __________________________________________________
- Health Conditions (7) __________________________________________________
- Additional Questions (8) __________________________________________________

In general, how would you rate the scoring of the BISQ elements across all relevant sections?

|  | Extremely difficult (1) | Somewhat difficult (2) | Neither easy nor difficult (3) | Somewhat easy (4) | Extremely easy (5) |
| --- | --- | --- | --- | --- | --- |
| Injury experience (Column A) (1) |  |  |  |  |  |
| Injury count (Column A) (2) |  |  |  |  |  |
| Hospitalization/ER experience (Column C) (3) |  |  |  |  |  |
| Hospitalization/ER count (Column C) (4) |  |  |  |  |  |
| Loss of consciousness (Column B/D) (5) |  |  |  |  |  |
| Alteration of consciousness (Column B/D) (6) |  |  |  |  |  |
| Date(s) of occurrence (Column B/D) (7) |  |  |  |  |  |

What do you like about the BISQ? [Open text response]

What do you dislike about the BISQ? [Open text response]

What changes (if any) do you think would improve the BISQ for use with people involved in the criminal legal system? [Open text response]

End of Block: BISQ

Start of Block: Post Discharge/Outpatient Treatment

How would you rate the administration of the Post Discharge/Outpatient Treatment measure?

- Extremely difficult (1)
- Somewhat difficult (2)
- Neither easy nor difficult (3)
- Somewhat easy (4)
- Extremely easy (5)

What do you like about the Post Discharge/Outpatient Treatment measure? [Open text response]

What do you dislike about the Post Discharge/Outpatient Treatment measure? [Open text response]

What changes (if any) do you think would improve the Post Discharge/Outpatient Treatment measure for use with people involved in the criminal legal system? [Open text response]

End of Block: Post Discharge/Outpatient Treatment

Start of Block: TBI Symptoms & Signs

How would you rate the administration of the TBI Symptoms & Signs measure?

- Extremely difficult (1)
- Somewhat difficult (2)
- Neither easy nor difficult (3)
- Somewhat easy (4)
- Extremely easy (5)

What do you like about the TBI Symptoms & Signs measure? [Open text response]

What do you dislike about the TBI Symptoms & Signs measure? [Open text response]

What changes (if any) do you think would improve the TBI Symptoms & Signs measure for use with people involved in the criminal legal system? [Open text response]

End of Block: TBI Symptoms & Signs

Start of Block: Screening Tools

How would you rate the administration of the Screening Tools?

- Extremely difficult (1)
- Somewhat difficult (2)
- Neither easy nor difficult (3)
- Somewhat easy (4)
- Extremely easy (5)

What do you like about the Screening Tools? [Open text response]

What do you dislike about the Screening Tools? [Open text response]

What changes (if any) do you think would improve the Screening Tools for use with people involved in the criminal legal system? [Open text response]

End of Block: Screening Tools

Start of Block: PCL-C

How would you rate the administration of the PTSD Checklist (PCL-C)?

- Extremely difficult (1)
- Somewhat difficult (2)
- Neither easy nor difficult (3)
- Somewhat easy (4)
- Extremely easy (5)

How would you rate the scoring of the PTSD Checklist (PCL-C)?

- Extremely difficult (1)
- Somewhat difficult (2)
- Neither easy nor difficult (3)
- Somewhat easy (4)
- Extremely easy (5)

What do you like about the PTSD Checklist (PCL-C)? [Open text response]

What do you dislike about the PTSD Checklist (PCL-C)? [Open text response]

What changes (if any) do you think would improve the PTSD Checklist (PCL-C) for use with people involved in the criminal legal system? [Open text response]

End of Block: PCL-C

Start of Block: Behavioral History

How would you rate the administration of the Behavioral History measure?

- Extremely difficult (1)
- Somewhat difficult (2)
- Neither easy nor difficult (3)
- Somewhat easy (4)
- Extremely easy (5)

How would you rate the scoring of the Behavioral History measure?

- Extremely difficult (1)
- Somewhat difficult (2)
- Neither easy nor difficult (3)
- Somewhat easy (4)
- Extremely easy (5)

What do you like about the Behavioral History measure? [Open text response]

What do you dislike about the Behavioral History measure? [Open text response]

What changes (if any) do you think would improve the Behavioral History measure for use with people involved in the criminal legal system? [Open text response]

End of Block: Behavioral History

Start of Block: RNS

How would you rate the administration of the Rehabilitation Needs Survey?

- Extremely difficult (1)
- Somewhat difficult (2)
- Neither easy nor difficult (3)
- Somewhat easy (4)
- Extremely easy (5)

Please describe...

- Your preferred approach to administer the RNS items (1) __________________________________________________
- Why you preferred this approach (2) __________________________________________________

The final Rehabilitation Needs Survey item ("Adjusting to life in the community") was adapted for use with people involved in the criminal legal system. Does this item appear to capture a meaningful aspect of participants' needs?

- Strongly disagree (1)
- Somewhat disagree (2)
- Neither agree nor disagree (3)
- Somewhat agree (4)
- Strongly agree (5)

What do you like about the Rehabilitation Needs Survey? [Open text response]

What do you dislike about the Rehabilitation Needs Survey? [Open text response]

What changes (if any) do you think would improve the Rehabilitation Needs Survey for use with people involved in the criminal legal system? [Open text response]

End of Block: RNS

Start of Block: Satisfaction with Life Scale

How would you rate the administration of the Satisfaction with Life Scale?

- Extremely difficult (1)
- Somewhat difficult (2)
- Neither easy nor difficult (3)
- Somewhat easy (4)
- Extremely easy (5)

How would you rate the scoring of the Satisfaction with Life Scale?

- Extremely difficult (1)
- Somewhat difficult (2)
- Neither easy nor difficult (3)
- Somewhat easy (4)
- Extremely easy (5)

What do you like about the Satisfaction with Life Scale? [Open text response]

What do you dislike about the Satisfaction with Life Scale? [Open text response]

What changes (if any) do you think would improve the Satisfaction with Life Scale for use with people involved in the criminal legal system? [Open text response]

End of Block: Satisfaction with Life Scale

Start of Block: Justice-Involvement Status

How would you rate the administration of the Justice-Involvement Status measure?

|  | Extremely difficult (1) | Somewhat difficult (2) | Neither easy nor difficult (3) | Somewhat easy (4) | Extremely easy (5) |
| --- | --- | --- | --- | --- | --- |
| Type (Core) (1) |  |  |  |  |  |
| Jurisdiction (Supplemental) (2) |  |  |  |  |  |
| Start date(s) (Supplemental) (3) |  |  |  |  |  |
| End date(s) (Supplemental) (4) |  |  |  |  |  |

How would you rate the scoring of the Justice-Involvement Status measure?

- Extremely difficult (1)
- Somewhat difficult (2)
- Neither easy nor difficult (3)
- Somewhat easy (4)
- Extremely easy (5)

What do you like about the Justice-Involvement Status measure? [Open text response]

What do you dislike about the Justice-Involvement Status measure? [Open text response]

What changes (if any) do you think would improve the Justice-Involvement Status measure? Please consider both your experience while administering this measure, as well as participants' statements, emotions, and other reactions to this measure when responding. [Open text response]

End of Block: Justice-Involvement Status

Start of Block: Completion Codes

How would you rate the use of the Completion Codes on the Coversheet?

- Extremely difficult (1)
- Somewhat difficult (2)
- Neither easy nor difficult (3)
- Somewhat easy (4)
- Extremely easy (5)

How would you rate the following Completion Codes?

|  | Recommend keeping (1) | Recommend modifying (2) | Recommend removing (3) | No Opinion (4) |
| --- | --- | --- | --- | --- |
| 01 - Test Administered in Full - Results Valid (1) |  |  |  |  |
| 02 - Test Administered in Full - Results Pending (2) |  |  |  |  |
| 03 - Test Administered in Full - Results Invalid (3) |  |  |  |  |
| 11 - Test Attempted BUT Not Completed (Due to Cognitive/Neurological Reasons) (4) |  |  |  |  |
| 12 - Test Attempted BUT Not Completed (Due to Other Medical reasons) (5) |  |  |  |  |
| 13 - Test Attempted BUT Not Completed (Due to Interactive Complexity) (6) |  |  |  |  |
| 14 - Test Attempted BUT Not Completed (Participant Declined to Continue) (7) |  |  |  |  |
| 15 - Test Attempted BUT Not Completed (Interrupted Administration) (8) |  |  |  |  |
| 16 - Test Attempted BUT Not Completed (Other Logistical Reasons) (9) |  |  |  |  |
| 17 - Test NOT Attempted (Provide Information) (10) |  |  |  |  |
| 20 - Other (Provide Information) (11) |  |  |  |  |

What modifications (if any) would you recommend to the Completion Codes? [Open text response]

Why would you recommend removing the selected Completion Codes? [Open text response]

What additions (if any) would you recommend to the Completion Codes? [Open text response]

End of Block: Completion Codes

Start of Block: Other

Do you have any other suggestions or modifications that you think would improve the Brain Health Project? [Open text response]

End of Block: Other
